# The Influence of Specific Bioactive Collagen Peptides on Knee Joint Discomfort in Young Physically Active Adults: A Randomized Controlled Trial

**DOI:** 10.3390/nu13020523

**Published:** 2021-02-05

**Authors:** Denise Zdzieblik, Judith Brame, Steffen Oesser, Albert Gollhofer, Daniel König

**Affiliations:** 1 Department for Nutrition, Institute for Sports and Sports Science, University of Freiburg, Schwarzwaldstr. 175, 79117 Freiburg, Germany; Denise.Zdzieblik@sport.uni-freiburg.de (D.Z.); AG@sport.uni-freiburg.de (A.G.); 2 CRI, Collagen Research Institute, Schauenburgerstr 116, 24118 Kiel, Germany; steffen.oesser@cri-mail.org; 3 Centre of Sports Science, Department for Sports, Nutrition and Health, University of Vienna, Auf der Schmelz 6, Vienna 1150, Austria; Daniel.Koenig@univie.ac.at; 4 Faculty of Life Sciences, Department for Sports, Nutrition and Health, University of Vienna, Althanstrasse 14, Vienna 1090, Austria

**Keywords:** activity-related knee pain, collagen peptides, VAS, range of motion, pain reduction

## Abstract

First evidence indicates that the supplementation of specific collagen peptides is associated with a significant reduction in activity-related joint pain in young adults. The purpose of the current investigation was to confirm the efficacy of the same collagen peptides in a comparable study population. In total, 180 active men and women aged between 18 and 30 years with exercise-related knee pain but no diagnosed joint disease completed the trial over a period of 12 weeks. Participants were randomly assigned to the group receiving 5 g of specific collagen peptides (CP-G) or to the placebo group (P-G). For the primary outcome, changes in pain during or after exercise from pre- to post-intervention were assessed by the participants using the Visual Analog Scale (VAS). These changes were additionally evaluated by the examining physician by means of anamnesis and physical examination of the affected knee joint. As secondary outcomes, pain under resting conditions and after 20 squats were compared between the study groups. In addition, the mobility of the knee joint and the use of alternative therapies (e.g., ointments or physiotherapy) were recorded. The supplementation of specific collagen peptides derived from type I collagen with a mean molecular weight of 3 kDa led to a significantly (*p* = 0.024) higher reduction of exercise-induced knee pain (−21.9 ± 18.3 mm) compared with the placebo group (−15.6 ± 18.5 mm). These findings were consistent with the physician’s evaluation (−23.0 ± 19.2 mm vs. −14.6 ± 17.9 mm, *p* = 0.003). The decrease in pain under resting conditions and after squats did not significantly differ between the groups, as only a small number of participants suffered from pain under these conditions. Due to the clinically unremarkable baseline values, the mobility of the knee joint did not change significantly after the intervention. In conclusion, the current investigation confirmed that the oral intake of bioactive collagen peptides used in the current investigation led to a statistically significant reduction of activity-related joint pain in young active adults suffering from knee joint discomfort.

## 1. Introduction

In physically active subjects, pain, injury and diseases of the musculoskeletal system are a relatively common complication causing impaired performance and reduced training gain [1,2,3]. In addition, in younger years, abnormal pressure due to sudden twisting movements or changes in direction can potentially injure joints, tendons and ligaments. The hyaline cartilage consists mainly of pressure-compensating proteoglycans and collagen fibers. Collagen type I and II are the most important structural and functional components of the extracellular matrix of tendons, ligaments and cartilage [4]. Quantitative and qualitative changes in the matrix structure (e.g., a decreased collagen content) are detected by tissue-specific cells, which trigger the stimulation of anabolic and catabolic processes to regulate the physiological extra cellular matrix macromolecule composition. If this homeostasis is disturbed by a change in the content of collagen, a reduced mechanical tissue load occurs [5,6,7,8]. Consequently, long-term stress promotes the development of degenerative diseases if pain and aches are ignored or left untreated [9,10,11]. An increased risk of anterior cruciate ligament rupture [12] or shoulder dislocation [13] is also attributed to an impaired architecture of the connective tissue. Although most complaints are related to sport, non-athletes may also sustain recurrent overuse injuries [10,11,14,15,16].

Acute degradation of connective tissue in tendons, cartilages and ligaments must be repaired, which is associated with a cascade of inflammation and pain [17]. Long-term biomechanical stress, high loading impact on the joint or sudden unphysiological movements (e.g., rapid stopping or changes of direction) can lead to acute or chronic overuse of the joint and, if not treated, to injuries or chronic degenerative diseases. The cause—regardless of whether muscles, tendons or cartilage tissue are affected—is a disproportion between load and load tolerance of the tissue [18]. Excessive training, inadequate recovery periods, incorrect techniques or movement sequences as well as constant competitive stress can be extrinsic factors that potentially increase the incidence of stress-induced injuries in every age group. In addition, intrinsic factors such as loss of flexibility or anatomical deformities (leg length differences or misaligned joints) potentially predispose athletes to overuse injuries [11].

Therapeutic approaches to joint diseases mainly focus on reducing symptoms and preventing subsequent ailments to maintain mobility and quality of life [1].

In this context, the efficacy and benefits of collagen peptides on joint health have been investigated in several scientific studies.

Current data from preclinical studies indicate that collagen peptides have a particularly high absorption rate [19,20,21,22]. Due to their low molecular weight and the high proportion of proline and hydroxyproline, collagen peptides show a high resistance to intestinal digestion and higher transport efficiency [19,23,24,25].

It has been demonstrated that collagen-derived peptides accumulate in the cartilage tissue, where they can stimulate the chondrocytes to synthesize cartilage extracellular matrix molecules and counteract progressive tissue degeneration [26,27,28,29,30]. The increased content of proteoglycan—an essential component of cartilage in the human knee joint—was a main outcome [31] of a pilot study by McAlindon et al. (2011).

In a series of clinical studies, pain and joint function in osteoarthritic patients were clearly improved by the daily administration of collagen peptides over a period of three to six months, whereas in other trials the effect on pain and joint mobility was less pronounced or limited to a subgroup [31,32,33,34,35,36,37]. Since a short-term increase in degradation can be assumed during or immediately after exercise [5], the positive effect of collagen peptides was also tested in activity-related joint complaints involving non-diseased subjects. According to the results of Clark et al. (2008), the daily intake of 10 g of collagen peptides over 24 weeks supports joint health and reduces pain, with the main effect in the knee joint [38]. A follow-up study by Zdzieblik et al. (2017) confirmed the results of Clark et al. (2008). The results imply that the intake of 5 g of specific collagen peptides for 12 weeks is sufficient to significantly reduce pain intensity during physical activity. Based on the survey of the participants, it can be assumed that medical treatment options such as drugs or physiotherapy could be reduced by the pain-relieving effect of collagen peptides [39].

Currently, limited data are available on the effect of collagen peptides on functional knee joint discomforts. To ensure the reliability of the previously published studies, the current trial was conducted to confirm the previous results in a larger group of individuals suffering exclusively from knee joint pain.

## 2. Materials and Methods

### 2.1. Study Design and Participants

This investigation was designed as a single-center, prospective, randomized, double-blind, placebo-controlled trial conducted at the University of Freiburg, Germany. In total, 218 healthy physically active (sports activities of more than 3 h a week) men and women aged between 18 and 30 years with activity-related functional knee joint pain (≥20 mm VAS scale) were randomized within the last 18 months. Based on previous results of a clinical trial involving physically active adults suffering from activity-related joint complaints [39], the sample size for the study was calculated using G*Power (University of Düsseldorf, Germany). Physically active adults were not eligible to participate if they were diagnosed with injuries, osteoarthritis, rheumatoid arthritis or other knee joint diseases. Reported intra-articular injections or the ingestion of glucosamine, chondroitin, hyaluronic acid or collagen products in the last 6 months were also defined as exclusion criteria. In addition, unstable weight (more than ±5 kg change within 3 months) and changes in eating habits led to exclusion of the participants screened. Participation in the study was also not possible if the participants suffered from extreme pain symptoms that required high-dose analgesic therapy over a longer period (>2 weeks) or intra-articular injection treatment.

The study was approved by the Ethics Committee of the University of Freiburg (ETK: 205/16) and registered in the German Clinical Trials Register (DRKS00015522). After written informed consent was obtained, participants were assigned to the group receiving collagen peptides (CP-G) or the placebo group (P-G) using a web-based random number generator [40]. To guarantee the blindness of the physician and the participants, the sachets containing the investigational products were absolutely identical in appearance and the products were equal in flavor and texture. Additionally, the responsible statistician was blinded during the analysis procedures. The data were not unblinded until data collection and database lock were both completed.

### 2.2. Efficacy Outcomes

Changes in pain intensity during activity after 12 weeks of supplementation, which were assessed by the study participants and attending physician, were defined as primary endpoints. The differences between the CP-G and the P-G were compared for this purpose.

Pain intensity was quantified using a visual analog scale (VAS), a validated measuring instrument for quantitative assessment between 0 (“no pain”) and 100 (“worst pain”) with intermediate levels. The distance between “no pain” and the point marked by the participant is given as the VAS score in mm [41].

During the baseline (T0) and the final (T12) visit, the physician examined the affected knee to exclude structural knee complaints and to determine the functionality for VAS assessment. The following tests were performed:

- McMurray test (in conjunction with Bragard) for meniscus injury [42]
- Steinmann test (I and II) to ascertain instability of the medial and lateral collateral ligament [43]
- The drawer test (Butler et al. 1980) for the anterior and posterior cruciate ligament [44]

As a secondary outcome, the participants assessed pain during 20 squats without additional weights and pain at rest that was not related to any previous activity.

Furthermore, changes in knee joint mobility (flexion and extension) and the use of treatments (physiotherapy, massage, etc.) were also considered as secondary endpoints. The start of new treatment options during the intervention led to exclusion from further participation.

To measure the range of motion of the knee joint, a three-digit code indicates the extent of movement in degrees. Starting from the zero position (basic position of the knee joint when standing upright), the maximum extension (first digit) and the maximum flexion (third digit) are recorded. If the zero position cannot be reached or passed, the zero is in the first position instead of the second, and the deficit is listed as the second digit [45].

### 2.3. Investigational Products

A specific mixture of porcine bioactive collagen peptides (FORTIGEL^®^, GELITA AG, Eberbach, Germany) with a high safety (GRAS status) was used for this study. The peptides derived from a special hydrolysis of type I collagen with a mean molecular weight of about 3 kDa and are characterized by the molecular weight fraction and the amino acid sequence. The reference product was a placebo containing maltodextrin (Walter GmbH, Olpe, Germany). All test products were packed in single sachets containing a daily dose of 5 g. The powders had to be dissolved in 250 mL of water at room temperature and ingested once daily for 12 weeks.

To check compliance, supplements not used were collected from the subjects at the final visit. In addition, the supplementation was documented in the compliance calendar.

### 2.4. Statistical Analysis

All data are presented as mean ± standard deviation (SD) in tables and mean ± standard error (SEM) in figures. SPSS statistics (IBM SPSS Statistics for Windows, Version 25.0. Armonk, NY: IBM Corp.) was used for all statistical analyses. All tests in the descriptive analysis were performed as two-sided tests, and the significance level was set at α = 0.05.

Data distribution was investigated using a Shapiro–Wilk test. In the case of normal distribution, the homogeneity of baseline values between the study groups was checked by an independent t-test. Otherwise, the Mann–Whitney U test was used. Dichotomous baseline values were tested by the chi-square test.

The mean differences obtained from CP-G and P-G were compared using a linear mixed model (LMM) for continuous variables. The factors were treatment (collagen peptides and placebo) and time (pre- and post-intervention levels). The frequencies of alternative treatment options were listed and compared between CP-G and P-G using the chi-square test.

The changes in VAS scores and knee joint mobility during the intervention period within the groups were analyzed using the paired sample t-test or the Wilcoxon signed-rank test when it could not be assumed that the data were normally distributed. The comparison of dichotomous data within the groups was performed using the paired McNemar chi-square test.

The Bonferroni–Holm correction was applied to the α level to control the total type I error rate, since 2 primary endpoints with no hierarchy were defined. The smallest *p* value was compared against α/2 (= 0.025) and the second smallest against α/1 (= 0.05).

As a magnitude of the difference between groups, the effect sizes were calculated from differences in means between groups at the end of the investigation (Cohen’s d).

For the exploratory part of the study, gender-related differences of the same parameters obtained from both groups were also compared using LMM for continuous variables. The factors were treatment (collagen peptides and placebo) and time (pre- and post-intervention levels). Furthermore, the gender was included as a covariate in the analysis.

## 3. Results

### 3.1. Subjects

**Figure 1:**
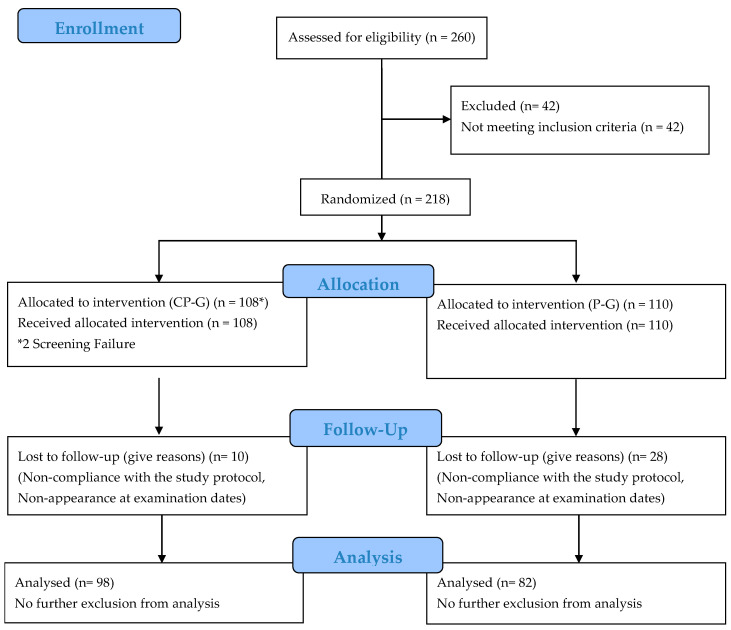
Flow chart of subject recruitment, randomization and follow up.

Of the 260 subjects screened, 218 were randomized. In total, 180 completed the trial. The per-protocol population (PP population) included 180 subjects (98 subjects in the CP-G and 82 in the P-G). The study dropouts are shown in Figure 1. The dropouts were related to non-compliance with the study protocol. None of the dropouts were related to any side effects or adverse events caused by taking the collagen peptide supplement or placebo. No adverse events were noted and, in particular, no pathological findings were observed in routine testing.

The baseline data of the study participants are summarized in Table 1. No statistically significant differences for any demographic result were observed between the two study groups in the PP population at the beginning of the study (Table 1). Although the total population analyzed (*p* = 0.004) contained significantly more women than men, there were no significant differences in the gender distribution in the groups.

### 3.2. Change in Knee Pain and Range of Motion

**Figure 2:**
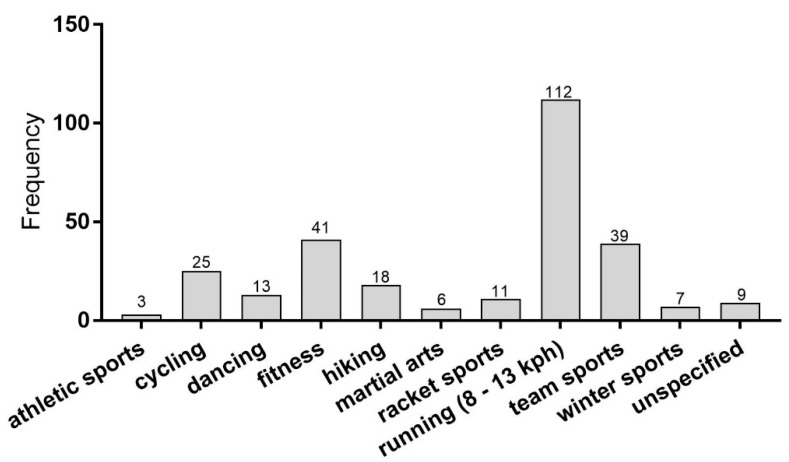
Absolute number of pain-causing activities. Data show the absolute case number (multiple answers possible). kph, kilometers per hour.

The anamnesis of the initial physical examination indicated that 32.2% of the participants in the evaluated study population had knee joint pain directly during activity. In 17.2% of the participants, the pain occurred immediately after the activity. Half of the athletes had knee joint pain during and after physical activity. Forty-four percent reported knee pain in both knees, while 56% suffered from knee pain either in the left (25%) or right (31%) knee. According to the medical history, endurance exercises, team sports and fitness training were the main activities leading to knee pain (Figure 2). In this study, an inadequate or overloading stress was reported as reason for the development of activity-related knee pain in 136 cases (75.6%). Anatomical deformities (leg length differences or misaligned joints) predisposed 44 (24.4%) participants to the development of pain related to physical exercises.

The baseline data of pain assessment and range of motion are summarized in Table 2. No significant baseline differences were found between the study groups. The current investigation showed a statistically significant reduction in pain during activity, pain during squats and pain at rest in both groups, when assessed by the participant. In addition, the pain during activity assessed by the physician decreased in both groups by a statistically significant level (Table 2).

Based on the VAS scores, the CP-G exhibit a statistically significant greater improvement in activity-related joint pain assessed by the subjects (*p* = 0.024) and physician (*p* = 0.003) than the P-G (Table 2). According to the physician’s assessment, these changes in activity-related pain had a medium effect, when taking the changes in the P-G as reference (d = −0.453). The evaluation n of the subjects showed also a clinically relevant effect compared with the P-G (d = −0.342).

**Figure 3:**
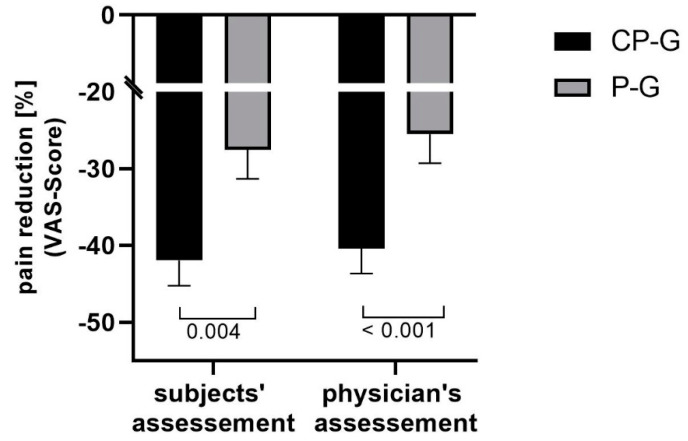
Relative changes in activity-related pain (VAS Score). Data are shown as mean ± SEM.

Taking into account the size of the individual differences in the VAS scores at the beginning of the study, the relative mean changes in activity-related pain were statistically significantly different between the study groups for the subjects’ (*p* = 0.004) and physician’s (*p* < 0.001) assessment (Figure 3).

The significance of the relative changes in pain during the activities assessed by the subjects and the physician was confirmed by the medium effect sizes (d = −0.432) and (d = −0.449).

The changes in pain under resting conditions or after performing squats were not statistically significantly different between the study groups. As none of the study parti-cipants showed restricted mobility of the knee joint at the beginning of the study, no changes in knee extension and flexion could be observed in either study group during the 12-week treatment. The data show no statistically significant differences between collagen peptide treatment and the placebo at the end of the trial.

An intention-to-treat analysis including all 218 participants confirmed the results of the PP analysis. The LMM analysis revealed a statistically significant difference between the CP-G and P-G regarding changes in pain during activity, which were assessed by the subject (*p* = 0.031) and the physician (*p* = 0.003).

The results of the exploratory analysis revealed no statistically significant influence by the gender. The differences between groups with respect to activity-related pain assessed by the subjects and the physician remains statistically significant when the gender is included as covariate. In contrast, no statistically significant differences were observed between groups when comparing changes in pain during squats, under rest or the extension and flexion of the effected joint. Although the female participants stated a slightly higher initial pain during activity, squats and under resting conditions, no statistically significant baseline differences were observed between male and female in the CP-G and P-G. Furthermore, neither in the CP-G nor the P-G changes in the respective outcomes differed statistically significant between male and female participants (Table 3).

### 3.3. Additional Treatment Options

Table 3 summarizes the use of additional therapies in the PP population at the beginning and end of the study. The group difference at baseline level (*p* = 0.437) and after the intake of 5 g collagen peptides per day over 12 weeks (*p* = 0.981) was not statistically significant.

In both study groups analyzed, the need for additional therapies was statistically significantly reduced by the end of study (Table 4).

## 4. Discussion

The current study with young physically active men and women showed that the intake of 5 g of specific collagen peptides per day for twelve weeks can significantly reduce the intensity of activity-related knee joint pain assessed by the participants compared with placebo. These findings were confirmed by the physician’s assessment. The exploratory part of the study revealed that these changes were not influenced by the gender.

To date, there is only a limited number of intervention studies that investigated the pain-relieving effect of collagen peptides on functional joint complaints [38,39,46]. In a pilot study, the oral intake of collagen peptides resulted in an improvement in pain symptoms in stress-induced joint complaints. However, the lack of a control group makes it difficult to assess the individual effects of the dietary intervention on the results [46]. The results of Clark et al. (2008) showed that the daily intake of collagen peptides led to a reduction in pain in functional joint complaints. However, the adjustment to the level of significance of the multiple test samples showed no significant differences between the CP and placebo groups [38]. In the study by Zdzieblik et al. (2017), the daily intake of 5 g collagen peptides led to a reduction in stress-related knee pain in young active adults. After a twelve-week intervention, the differences in pain levels between the collagen peptide and placebo group were significant for activity-related pain according both, the assessment of the subjects and the physician [39]. At the same study dose (5 g per day) and duration (12 weeks), the pain reduction of 42% in the present study was comparable with the changes of 38% in the study by Zdzieblik et al. (2017). With the placebo as reference, the calculated effect size for the physician’s (d = −0.453) and the subject’s (d = −0.342) assessment was similar to the study by Zdzieblik et al. (2017), confirming the efficacy of 5 g collagen peptides daily as a therapeutic approach for activity-related knee joint pain.

Continuous stress or an insufficient recovery phase promotes the development of pain under resting conditions [18,47,48]. In the present study, the decrease in resting pain was not significant in the group comparison after the intervention as a potential consequence of low baseline values and a small number of participants suffering from pain at rest. Only 67 subjects (38%) reported pain at rest. These findings are consistent with the changes in pain after performing 20 squats. Only half of the study population had pain after squats with an initial VAS score of approximately 18 mm, which was lower than the inclusion criterion for activity-related pain. Since the impact of a standard bodyweight squat might be insufficient to evaluate joint pain under specific exercise conditions, higher loads or a standardized running test should be included in further investigations.

In the comparison of the study groups, knee-joint mobility did not improve significantly after the intervention. As the baseline values did not constitute any restrictions, improvements in joint mobility were not expected. Functional joint complaints are not characterized by a progressive, but possibly short-term increased cartilage degradation due to the exercise-induced stress on the knee joint [5]. Therefore, knee problems are not directly related to a functional impairment of the knee joint. In previous studies investigating the pain-relieving effect of collagen peptides in physically active men and women with exercise-induced pain, the participants also showed no restriction of knee movement [38,39].

In the present study, some subjects initially described instability of the knee joint. In these cases, improved joint stability was observed after 12 weeks of intervention with collagen peptides. Tendons and ligaments are important structures of the musculoskeletal system that determine the mobility and stability of the joints. Excessive mobility (hypermobility) due to increased laxity of the ligaments is associated with an increased risk of injury and osteoarthritis [49]. The influence of collagen peptides on the structure and functionality of tendons and ligaments is, therefore, a potential approach to alleviate stress-related knee joint problems.

The results of Dressler et al. (2018) showed improved ankle functionality, which was represented by significantly higher CAIT (Chronic Ankle Instability Tool) and FAAM-G (German version of Foot and Ankle Ability Measure) scores after the daily intake of 5 g specific collagen peptides with a mean molecular weight of 2 kDa over six months [50].

Reduced vascularization and consequently fewer tendon lesions due to oral ingestion of specific collagen peptides in combination with an eccentric exercise program has been one of the main outcomes of a clinical trial patients with Achilles Tendinopathy [51]. In addition, the Victorian Institute of Sports Assessment–Achilles (VISA-A) questionnaires—a specific, reliable and valid clinical measure of Achilles tendinopathy—showed a significant improvement in the areas of pain, function in daily life and physical activity through the intake of specific collagen peptides. Furthermore, the return to moderate running was a result of pain reduction [51].

According to the current state of research, the positive effect of collagen peptides in cartilage [26,27,28,29,31], tendon and ligament [52] tissue may be due to molecular biological processes such as the stimulation of elastin and collagen type I formation. Another explanatory approach is the inhibition of inflammatory and pain-inducing processes. In vivo experiments showed that the intake of collagen peptides led to a glycine receptor-mediated reduction in IL-6 release [53]. The suppression of TNF-α release with glycine has also been shown in preclinical studies [54,55]. By increasing the synthesis of extracellular macromolecules, the administration of collagen peptides could additionally reduce stress-related cartilage degradation and thus inhibit the downstream pro-inflammatory and pain-stimulating processes [56,57].

This trial has some limitations. With respect to the significant pain reduction in the P-G, the placebo might have an effect on how the participants perceived their condition (relieving pain) but had no impact on the activity-related knee joint pain. This assumption is supported by the smaller reduction in activity-related pain in the P-G, when taking the more objective assessment of the physician into account. To evaluate the efficacy of the used collagen peptides the differences between groups were defined as endpoints in the study. Furthermore, it cannot be excluded that changes in pain were influenced by slight changes in the activity level e.g., due to seasonal differences. Since the pain assessment depends on the participants’ subjective perceptions, biomechanical measurements, markers of inflammation or imaging techniques might complement the range of motion measurement to evaluate improvements in functionality and structural features in future studies.

First evidence suggests that the composition of collagen peptide preparations is heterogeneous with disparate pharmacological effects and that the efficacy of a collagen peptide mixture cannot extrapolated to other formulations [58]. Therefore, it must be emphasized that the presented results are only reliable for the collagen peptide product that was investigated in this study and cannot be transferred to other collagen products.

## 5. Conclusions

In this single-center, prospective, randomized, double-blind and placebo-controlled study, physically active young adults with activity-related knee joint pain were treated with a daily dosage of 5 g of specific collagen peptides or a placebo for 12 weeks.

The results of the primary endpoints of the study show a statistically significant reduction in “pain during activity” after oral treatment with specific collagen peptides compared with placebo, as assessed by the study participants and the physician.

Due to the small number of cases with noticeable pain at rest and after performing squats, the pain under these conditions was not statistically significantly affected by the daily intake of collagen peptides, nor did joint mobility change statistically due to full movability at baseline.

The current investigation confirmed that the specific collagen peptides used in the current study have a positive effect on knee joint pain during activity and may help to prevent the clinical manifestation of chronic degenerative joint diseases.

## Figures and Tables

**Table 1:** Baseline data (T0) for the PP population (*n* = 180).

	Total (*n* = 180)	CP-G (*n* = 98)	P-G (*n* = 82)	*p* Value
Age (years)	23.9 ± 2.90	23.9 ± 3.07	23.8 ± 2.70	0.885 *
Gender (male/female)	71/109	43/55	28/54	0.221 ^#^
Height (m)	1.74 ± 0.09	1.74 ± 0.09	1.73 ± 0.09	0.353 *
Body weight (kg)	67.5 ± 10.8	68.1 ± 11.4	66.8 ± 10.0	0.578 *
BMI (kg/m^2^)	22.3 ± 2.40	22.3 ± 2.53	22.2 ± 2.23	0.928 *
BP sys (mm Hg)	121.1 ± 12.1	121.4 ± 12.2	120.8 ± 12.0	0.476 *
BP dia (mm Hg)	74.2 ± 8.06	75.2 ± 8.71	73.1 ± 7.10	0.125 *

Data represent mean ± SD; * Mann–Whitney U test; ^#^ chi-square test.

**Table 2:** Knee pain and range of motion in the PP population.

	CP-G (*n* = 98)		P-G (*n* = 82)		*p* Value LMM
	T0	T12	T0	T12	
Pain during activity (subject) [mm]	55.3 ± 15.8	33.4 ± 21.9 ***	56.5 ± 15.0	40.8 ± 19.5 ***	**0.024**
Pain during activity (physician) [mm]	55.5 ± 13.5	32.6 ± 20.3 ***	55.4 ± 13.7	40.8 ± 18.1 ***	**0.003**
Pain during squat [mm]	16.6 ± 21.5	7.80 ± 16.0 ***	19.1 ± 21.7	10.1 ± 19.2 ***	0.961
Pain at rest [mm]	13.5 ± 22.0	6.68 ± 17.5 **	18.3 ± 25.8	9.83 ± 19.3 **	0.628
Extension [degree]	5.82 ± 4.16	5.93 ± 3.78	6.12 ± 4.07	6.22 ± 3.55	0.979
Flexion [degree]	146.6 ± 7.68	146.4 ± 8.91	147.4 ± 6.62	148.2 ± 8.35	0.301

Data represent mean ± SD. *p* value LMM, Significance between groups in Linear Mixed Model testing assessing treatment × time interaction. ** *p* < 0.01; *** *p* < 0.001 within the group from baseline to final examination (Wilcoxon signed-rank test). LMM: linear mixed model. Bold numbers represent statistical significance of primary endpoints

**Table 3:** Knee pain and range of motion in the male and female subgroups.

	Subgroup	CP-G (*n* = 98)		P-G (*n* = 82)		*p* Value LMM
		T0	T12	T0	T12	
Pain during activity (subject) [mm]	male	51.6 ± 14.7	29.2 ± 21.3 ***	54.0 ± 18.3	36.8 ± 22.9 ***	**0.028**
	female	58.0 ± 16.1	36.5 ± 22.0 ***	57.7 ± 13.0	42.9 ± 17.3 ***	
Pain during activity (physician) [mm]	male	52.5 ± 13.1	29.3 ± 20.3 ***	52.9 ± 15.6	35.9 ± 20.9 ***	**0.004**
	female	57.8 ± 13.4	35.0 ± 20.2 ***	56.7 ± 12.6	43.3 ± 16.1 ***	
Pain during squat [mm]	male	15.6 ± 22.0	6.67 ± 16.4 **	18.1 ± 23.0	8.75 ± 20.3 **	0.954
	female	17.4 ± 21.2	8.64 ± 15.8 **	19.6 ± 21.1	10.8 ± 18.8 **	
Pain at rest [mm]	male	11.7 ± 21.8	1.62 ± 7.87 **	16.3 ± 23.3	10.8 ± 21.0	0.608
	female	14.8 ± 22.3	10.5 ± 21.5	19.4 ± 27.1	9.33 ± 18.4 **	
Extension [degree]	male	5.38 ± 4.01	6.02 ± 3.87	7.11 ± 3.46	7.50 ± 2.89	0.933
	female	6.14 ± 4.28	5.86 ± 3.75	5.61 ± 4.29	5.56 ± 3.70	
Flexion [degree]	male	146.8 ± 8.51	145.9 ± 8.57	146.4 ± 7.06	147.1 ± 8.28	0.341
	female	146.4 ± 7.06	146.8 ± 9.21	147.9 ± 6.38	148.8 ± 8.40	

Data represent mean ± SD. *p* value LMM, Significance between groups in Linear Mixed Model testing assessing treatment × time interaction including gender as covariate. ** *p* < 0.01; *** *p* < 0.001 within the group from baseline to final examination (Wilcoxon signed-rank test). LMM: linear mixed model. Bold numbers represent statistical significance of primary endpoints considering the gender as covariate

**Table 4:** Additional therapies in the PP population.

	CP-G (*n* = 98)		P-G (*n* = 82)	
	T0	T12	T0	T12
In total	29	25	32	26
Ointments and gels	2	2	2	1
Bandages and orthosis	15	16	17	15
Physiotherapy	5	5	5	3
various therapies ^1^	7	2	8	7
*p* Value *	0.022		0.012	

Data represent absolute data; * McNemar chi-square test. ^1^ use of more than one therapy option.

## Data Availability

Data sharing not applicable.
